# Laser ablation assisted micropattern screen printed transduction electrodes for sensing applications

**DOI:** 10.1038/s41598-022-10878-6

**Published:** 2022-04-28

**Authors:** Muhammad Asif Ali Rehmani, Kartikay Lal, Ayesha Shaukat, Khalid Mahmood Arif

**Affiliations:** Department of Mechanical and Electrical Engineering, SF&AT, Massey University, Auckland, 0632 New Zealand

**Keywords:** Engineering, Electrical and electronic engineering, Mechanical engineering

## Abstract

In this work we present a facile method for the fabrication of several capacitive transduction electrodes for sensing applications. To prepare the electrodes, line widths up to 300 $\upmu$m were produced on polymethyl methacrylate (PMMA) substrate using a common workshop laser engraving machine. The geometries prepared with the laser ablation process were characterised by optical microscopy for consistency and accuracy. Later, the geometries were coated with functional polymer porous cellulose decorated sensing layer for humidity sensing. The resulting sensors were tested at various relative humidity (RH) levels. In general, good sensing response was produced by the sensors with sensitivities ranging from 0.13 to 2.37 pF/%RH. In ambient conditions the response time of 10 s was noticed for all the fabricated sensors. Moreover, experimental results show that the sensitivity of the fabricated sensors depends highly on the geometry and by changing the electrode geometry sensitivity increases up to 5 times can be achieved with the same sensing layer. The simplicity of the fabrication process and higher sensitivity resulting from the electrode designs is expected to enable the application of the proposed electrodes not only in air quality sensors but also in many other areas such as touch or tactile sensors.

## Introduction

Numerous fabrication techniques have been reported in literature to form the transduction schemes for sensors attaining new functionalities, superior device responses and capabilities. However, most of the techniques require intricate processes and costly facilities to fabricate such sensors. For instance, the conventional microelectromechanical systems (MEMs) photolithography process, which is a top-down approach for fabricating sensing electrodes, requires cleanroom and chemical etching process^1,2^. The overall process leads to chemical wastage, poses environmental issues^3–5^ and customization in the electrode design is often expensive as the product cost relies heavily on the scale and batch size of fabrication. Therefore, contactless printing and contact printing, not requiring the provision of a clean room, have attained interest recently for R&D activities. Contact printing is widely used in the paper industry and print media. The upside of these printing strategies is their high throughput with accuracies up to 50 $\upmu$m of printed features. Generally, all the contact printing methods use roll-to-roll technology to imprint the pattern on the substrate^6–8^. However, interconnect registration control, on account of tight tolerances and elastic nature of the substrate at high speed and pressure is intricate in nature. For large volume production the cost of printed features through roll-to-roll technology is cheaper than contactless printing method. However, for small production batches or customized imprints, the cost per item is a lot higher than contactless printing. Among contactless printing, inkjet printing has been widely used for printed electronic applications due to their low capital cost and pervasive availability. Moreover, compared to roll-to-roll printing technology the customized patterned printing can be done readily with the ability to print features or ink additively on the previously printed features. Thermal and piezoelectric inkjet techniques require formulation of ink, which needs to be compatible with the printing process. Ink often degrades in the thermal inkjet printing process if it is composed of material susceptible to thermal degradation, moreover high viscosity ink cannot be used with piezoelectric inkjet printers^9,10^. Screen printing for a simple lab-based R&D setup seems to be a possible solution for fabricating transduction electrodes at a much cheaper cost compared to the above-mentioned fabrication processes. Screen printing requires a stencil and although the process is simple, the low-cost customization of the transduction electrodes is a big issue and the process involves spreading a large amount of ink on the mesh. To circumvent the aforementioned issues, a simple laser ablation process for screen printing of conductive ink seems to a be an easier route for the fabrication of transduction electrodes. The laser ablation process from the commercial laser cutting machine not only provides facile implementation of transduction electrodes but also generates less ink waste when compared to the conventional screen printing of ink. In this work, printed capacitive structures to sense the electrochemical behaviour of the analyte are formed by laser ablation technique. The advantage of capacitive sensors is that they consume low energy, are less susceptible to radiation, have good sensitivity and provide fast response^11–16^. The most well-known design for measuring capacitive response is a parallel plate (PP) electrodes where the electrical terminals are isolated by a dielectric material^17,18^. For sensing applications and particularly in thin-film capacitive sensors, interdigitated electrodes (IDEs) are perhaps the most broadly utilized electrodes mostly due to their simple design, analytical and numerical modelling^19–22^.

**Figure 1:**
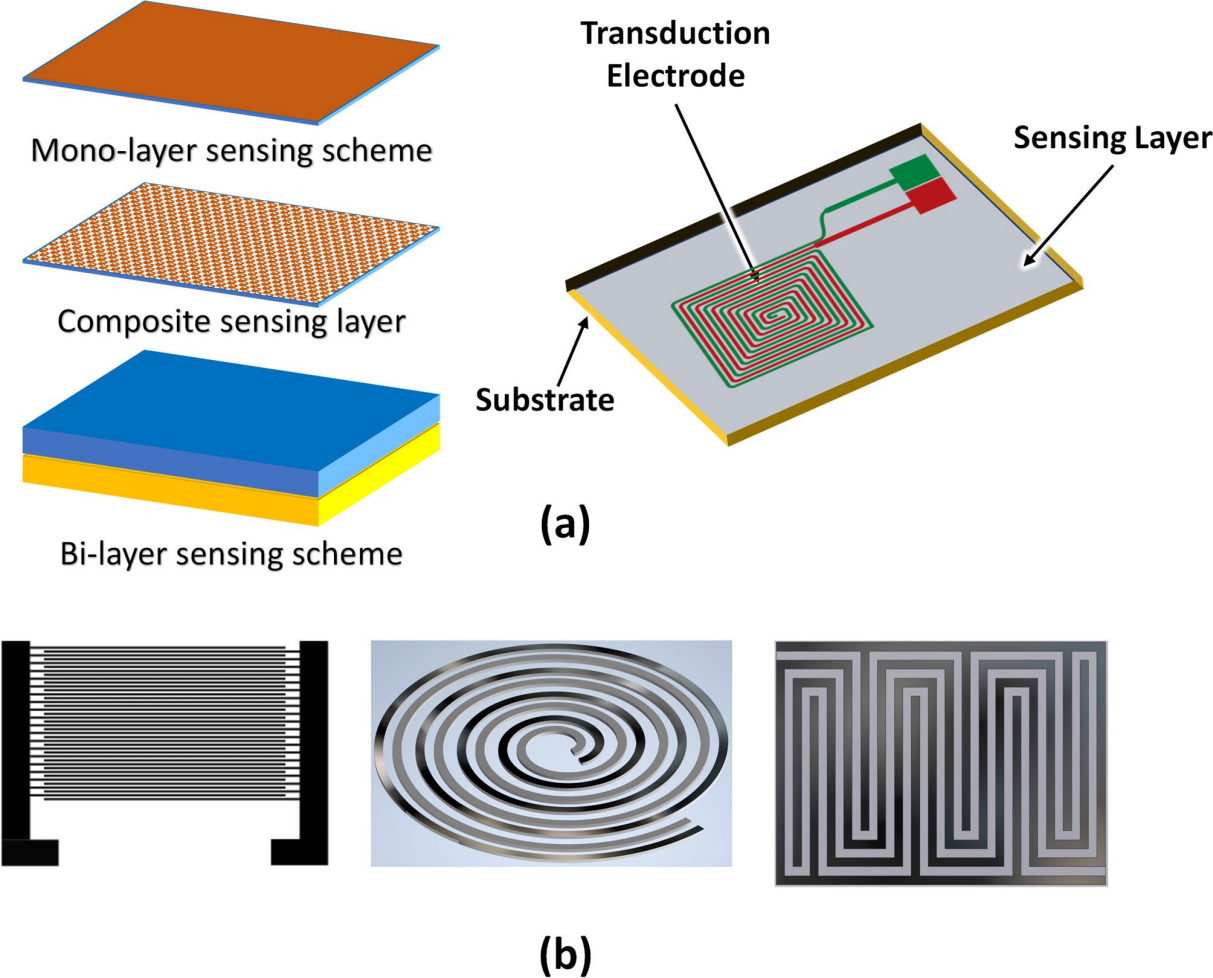
(**a**) Typical sensing mechanism of an electrochemical sensor. (**b**) Geometric shapes of transduction electrodes.

The basic components of an electrochemical sensor are the sensing layer, transduction electrodes and the substrate. The sensing layer attracts the analyte by undergoing chemi-adsorption, which generates the electrical signal sensed by the readout circuit. The rate of adsorption dictates the response of the sensor where the desorption cycle is attributed to the recovery of the sensing layer. The sensing layer can be a single layer, bilayer or composite layer. A typical sensor layout is presented in Fig. 1. Transduction electrodes can have different shapes or geometries such as indigitated or meander that provide the enhanced signal for capacitive and resistive sensing schemes^23^.

Capacitive sensing has been commonly used for humidity sensors with reference capacitors so to mitigate the drift due to thermal interference. However, these devices are complex due to the inclusion of additional components^24,25^. Other methods such as heating of substrate are also used to shorten or augment the recovery of such sensors^26^. Nevertheless, with proper selection of sensing layers, electrode geometry and suitable substrate a sensitive and highly responsive environmental sensor can be fabricated, which operates at room temperature with low or minimal sensor drift and without needing additional components^27,28^.

Fabricating the transduction schemes on the substrate requires intricate procedure and is often subjected to available resources. In the context of the prevailing COVID-19 pandemic situation most of the fabrication facilities are either non-accessible or closed^29^. In this scenario sensor fabrication techniques based on MEMs^30–32^, inkjet printing^33–35^ and contact printing^36–38^ methods can be expensive or unapproachable. However, a simple laser ablation technique by utilizing the desktop $\hbox {CO}_{\mathrm{2}}$ laser cutter can be used to fabricate the transduction schemes for realizing the environmental sensor through screen printing the conductive ink inside the ablated tracks.

## Results and discussion

**Figure 2:**
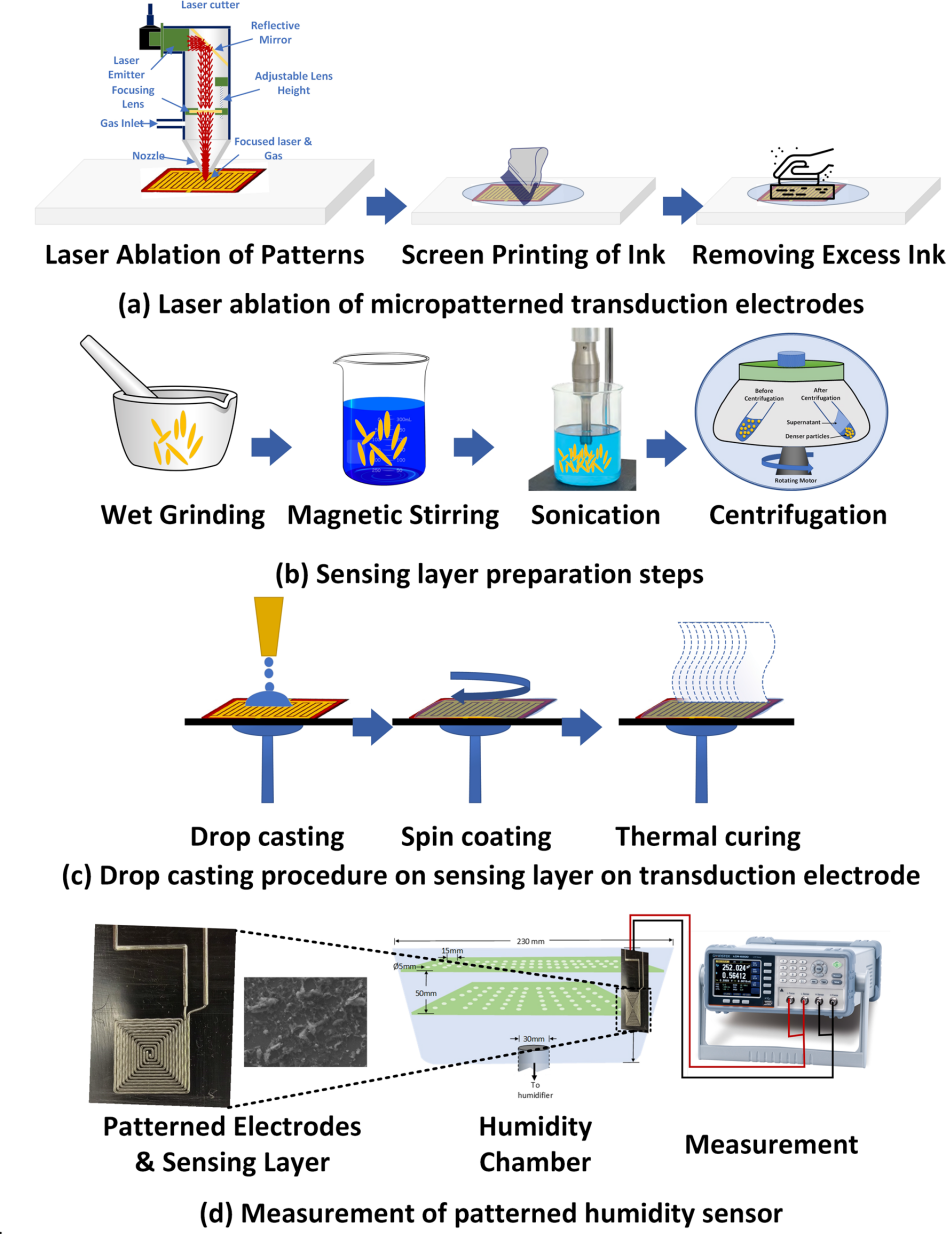
Steps for laser ablated micropatterned sensor fabrication.

A systematic methodology followed by pursuing the steps highlighted in Fig. 2, resulted in the fabrication of laser ablated micropatterned features having an average width resolution of around 290 $\upmu$m. Table 1 shows the variation in the experimental data of the patterned features.

**Table 1:** Experimental resolution of screen templating through laser engraving process.

Measure	Value (${{\upmu }}$m)
**Straight profiles electrode distance**	
Average	289
Minimum	287
Maximum	292
Standard deviation	2.1
**Curved profiles electrode distance**	
Average	377
Minimum	370
Maximum	383
Standard deviation	5.3
**Straight profile inter-electrode dpacing**	
Average	315
Minimum	313
Maximum	318
Standard deviation	2.1
**Curved profile inter-electrode spacing**	
Average	216
Minimum	208
Maximum	223
Standard deviation	6.1

**Figure 3:**
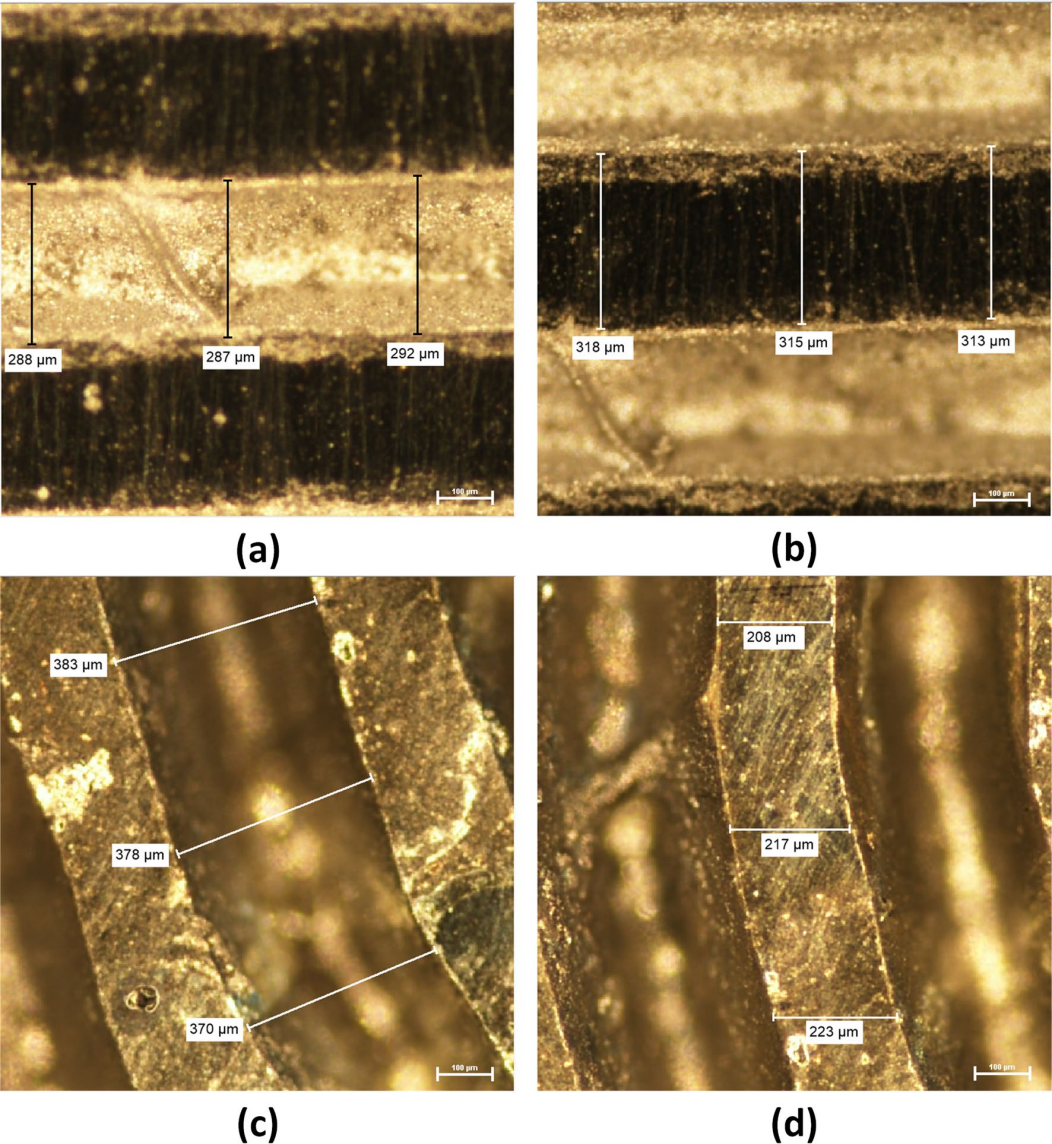
Optical images of laser ablated transduction features. (**a**) Straight profile, (**b**) straight profile electrode inter-spacing, (**c**) curved profile, and (**d**) curved profile electrode inter-spacing.

The microchannel is formed by the laser ablation process due to the absorption of energy induced by the laser beam. When the laser beam interacts with the workpiece it ablates the top surface of the workpiece. The rate of ablation depends upon the power, speed of the laser, wavelength of the radiation and material physical and optical properties. The resolution of the microchannel can be optimized by carefully selecting the parameters mentioned above. However, we have used the default ablation process parameters for ease of micropatterning and simplicity of fabricating micropattern, which can be utilized for sensing of humidity. In our experiments, we used 100% laser power and 100% speed for laser engraving on a 3 mm thick polymethyl methacrylate sheet. Figure 3 shows that for curved regions the resolution of patterned microchannel was degraded. The reason for degradation of the resolution is due to slower speed of the laser as compared to the straight feature. The X–Y stage of the laser scanning head uses successive straight-line interpolation and offsets to interpolate the next laser spot for a curved geometry on the workpiece. Due to this interpolation of points for a curved region the speed is slow, and more area is ablated due to the prolonged laser exposure at a particular position. Optical images of the curved and straight features depicting the differences in the line widths are shown in Fig. 3.

**Figure 4:**
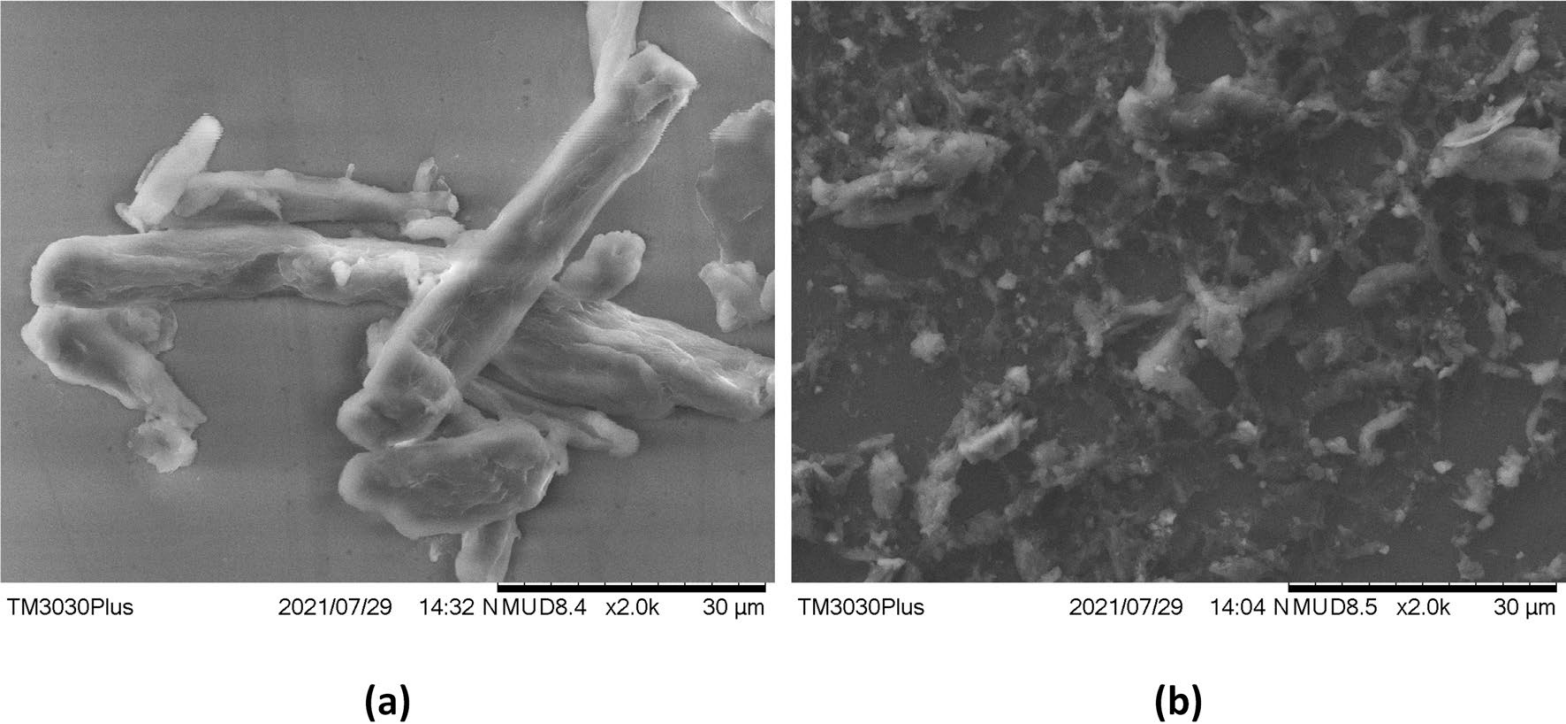
SEM images of cellulose decorated sensing layer. (**a**) Decorated cellulose before ultrasonication. (**b**) Decorated cellulose after ultrasonication.

Operations that were conducted to reduce the size of the suspended particles and to increase the activation sites can be seen from the scanning electron microscopy images in Fig. 4. The image in Fig. 4b shows the overall distribution of the cellulose after wet grinding, centrifugation and ultrasonication.

The sensing layer is based on biodegradable ink, which is mainly composed of cellulose, poly ethylene dioxythiophene: poly-styrene sulfonate (PEDOT:PSS)^39,40^ and polyvinylpyrrolidone (PVP) coated silver nanoparticles^41,42^. Silver nanoparticles (SNPs) are known for their antimicrobial properties as in medical applications silver catheters and silver coated catheters are used for slow-injection of solvent while providing antiseptic properties. Moreover, as the nanoparticles are coated with PVP, there are less chances of toxicity and the possibility of oxidation^43–45^. On the other hand, cellulose is a good natural insulator commonly used as a dielectric material. It has been incorporated into many applications as substrate for conductive and non-conductive applications^46,47^.

The sensing properties depend on the change in electrical properties of the sensing layer, which forms a uniform sensing region over the transduction electrodes. Once the sensing layer is exposed to humidity the porous structure of the cellulose decorated PEDOT:PSS and PVP coated SNP layer changes it’s capacitance during the adsorption and desorption cycles. The change in capacitance is then recorded for various humidity levels. All the sensors were tested with a starting relative humidity level of 50% as it was the prevailing ambient condition for conducting the humidity measurements. The measurements were taken with the help of GW INSTEK LCR-6000 Precision LCR Meter by sweeping the selectable frequencies between 100 Hz and 2 kHz as tabulated in Table 2. The formulation of the sensing layer with the addition of PVP coated SNPs has provided steric stability. The steric stability is evident in the SEM image as the cellulose fibres are well spread over the region. Not only the readings are stable but also the fluctuation of the capacitance value of the prepared sensors remains within standard deviation of 0.52 pF. In our experiments, when only the conductive PEDOT:PSS and cellulose mixture was spin-coated on the transduction electrodes, the capacitive reading from the fabricated sensors were not stable due to highly conductive PEDOT:PSS coated layer. The mixing procedure and addition of PVP coated SNP not only reduced the conductivity of the sensing layer but also provided anti-agglomeration property to the prepared ink for sensing. We noted that, on average, for all the transduction geometries the fluctuation in the capacitance was abrupt without the inclusion of PVP coated SNP steric stabilizer.

**Table 2:** Experimental resolution of screen templating through laser cutting process.

Capacitance of transduction geometries at 50% RH							
S. no.	Freq.	IDTs	Meander	Spiral	Swiss	Serpentine	Custom
1	100	15.87	19.43	17.30	19.65	21.23	23.10
2	200	15.52	19.09	16.88	19.21	20.84	22.75
3	300	15.28	18.95	16.71	18.90	20.58	22.48
4	400	15.24	18.63	16.70	18.77	20.43	22.34
5	500	15.14	18.66	16.56	18.60	20.32	22.32
6	750	15.14	18.42	16.39	18.22	20.31	22.39
7	1000	14.88	18.33	16.35	18.28	19.94	21.99
8	1250	14.73	18.18	16.36	18.14	19.80	21.73
9	1500	14.76	18.25	16.23	18.13	19.75	21.83
10	2000	14.68	18.16	16.16	18.00	19.60	21.75
Mean		15.12	18.61	16.56	18.59	20.28	22.27
SD		0.36	0.41	0.33	0.51	0.49	0.43
Schematic							

**Figure 5:**
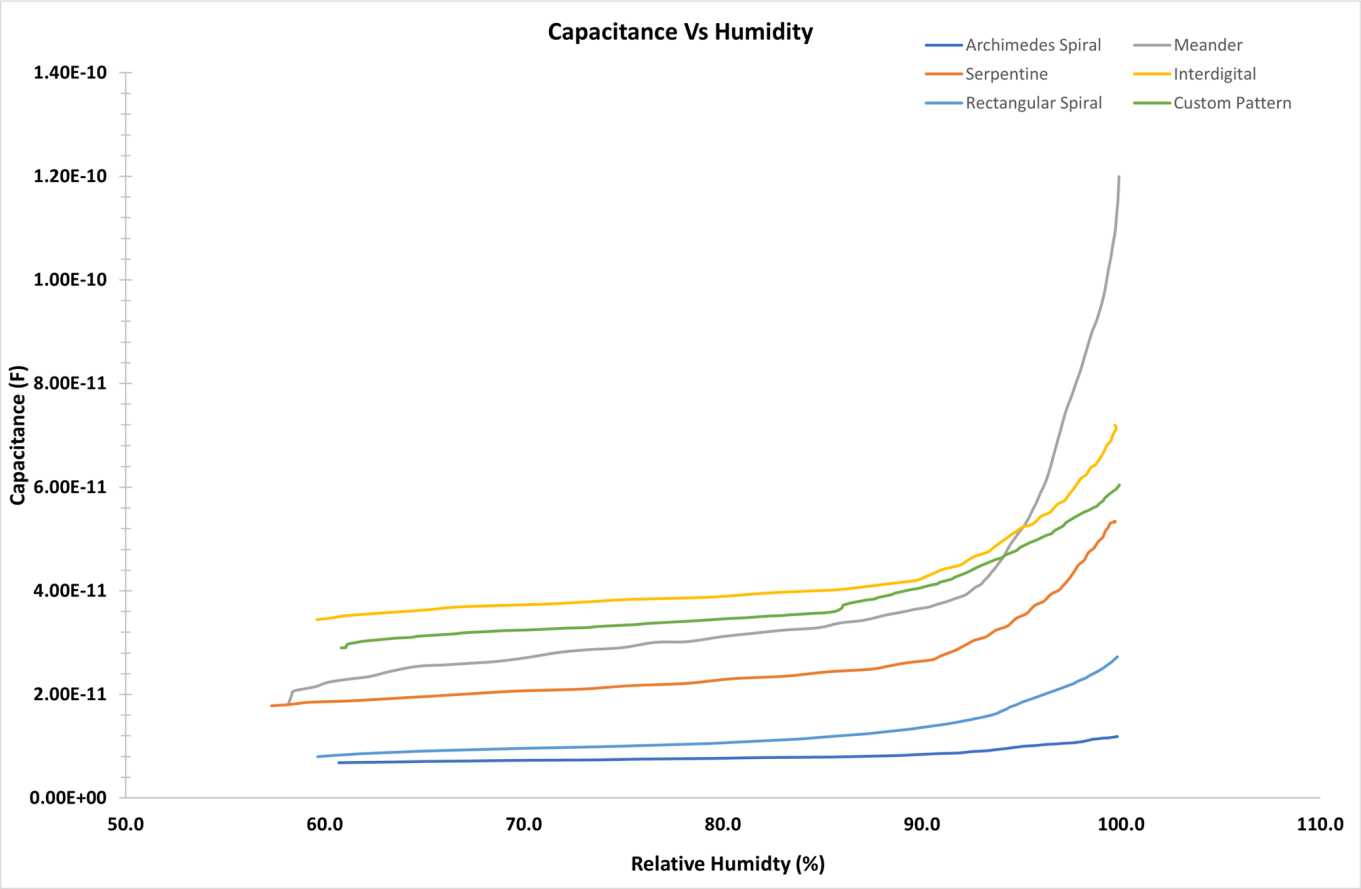
Humidity response of patterned sensors for (**a**) Archemedies spiral, (**b**) Meander, (**c**) Serpentine, (**d**) Interdigital, (**e**) Rectangular spiral, and (**f**) Custom design.

The results of the humidity response with the transduction geometries are highlighted in Fig. 5. The highest response was recorded with a meander electrode configuration having a sensitivity of 2.37 pF/%RH whereas the lowest response was from archenemies spiral configuration of 0.13 pF/%RH. At relative humidity level above 80% there was a sharp increase in capacitive response for meander electrode configuration as compared to the other geometric configuration. Serpentine, interdigital, and custom pattern has not only shown good sensitivity but a gradual increase in capacitive response with respect to relative humidity. Therefore, these configuration may be selected for practical ranges of humidity response. The reason for the variation in the transduction response is due to the difference in the density of the sensing electrodes and inflection points in the geometries. These changes result in the difference of electric field generated by the respective geometries thus exhibiting changes in the capacitance of each geometry.

The sensitivity of the sensor is defined as the ratio of difference of the capacitance at a specific relative humidity level designated by $\hbox {C}_{\mathrm{RH}}$ and base capacitance ($\hbox {C}_{\mathrm{RHo}}$) of the sensor divided by the base capacitance of the sensor. Equation 1 mathematically denotes the sensitivity of the sensor.

1 $$\begin{aligned} S=(C_{\mathrm{RH}}-C_{\mathrm{RHo}})/C_{\mathrm{RHo}} \end{aligned}$$

Table 3 shows the sensitivities of the different transduction schemes. It is evident from the sensitivity values that sensing gradient highly depends on the transduction geometry. For certain sensing application a same sensing layer can offer better result with a specific geometry.

**Table 3:** Sensitivity of the transduction geometries.

Sensitivity of fabricated sensors	
Transduction geometry	Sensitivity pF/%RH
Meander	2.37
Archimedes spiral	0.13
Serpentine	0.84
Interdigital	1.06
Rectangular spiral	0.31
Custom	0.88

**Figure 6:**
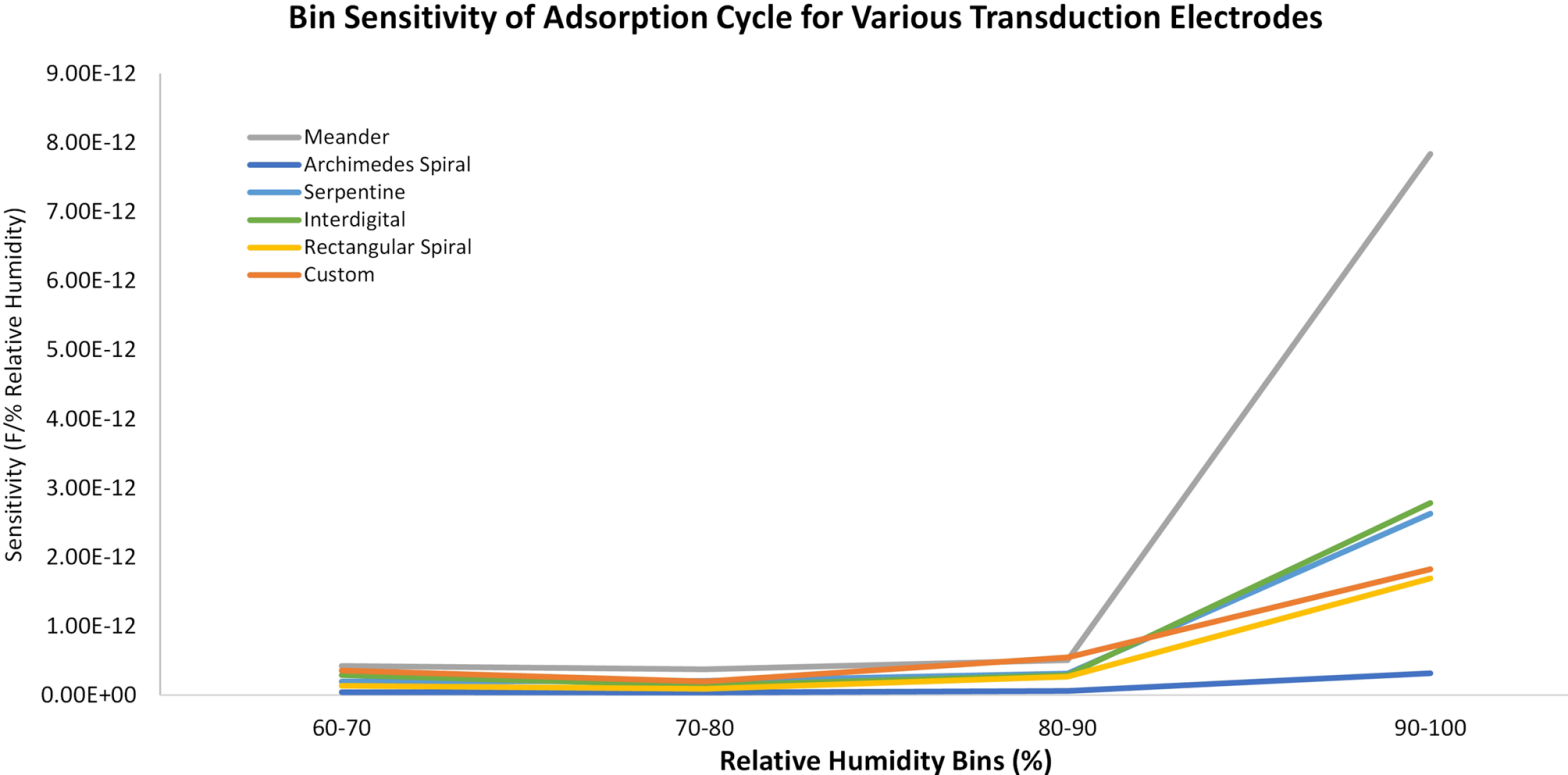
Bin sensitivities of all six transduction electrodes.

**Figure 7:**
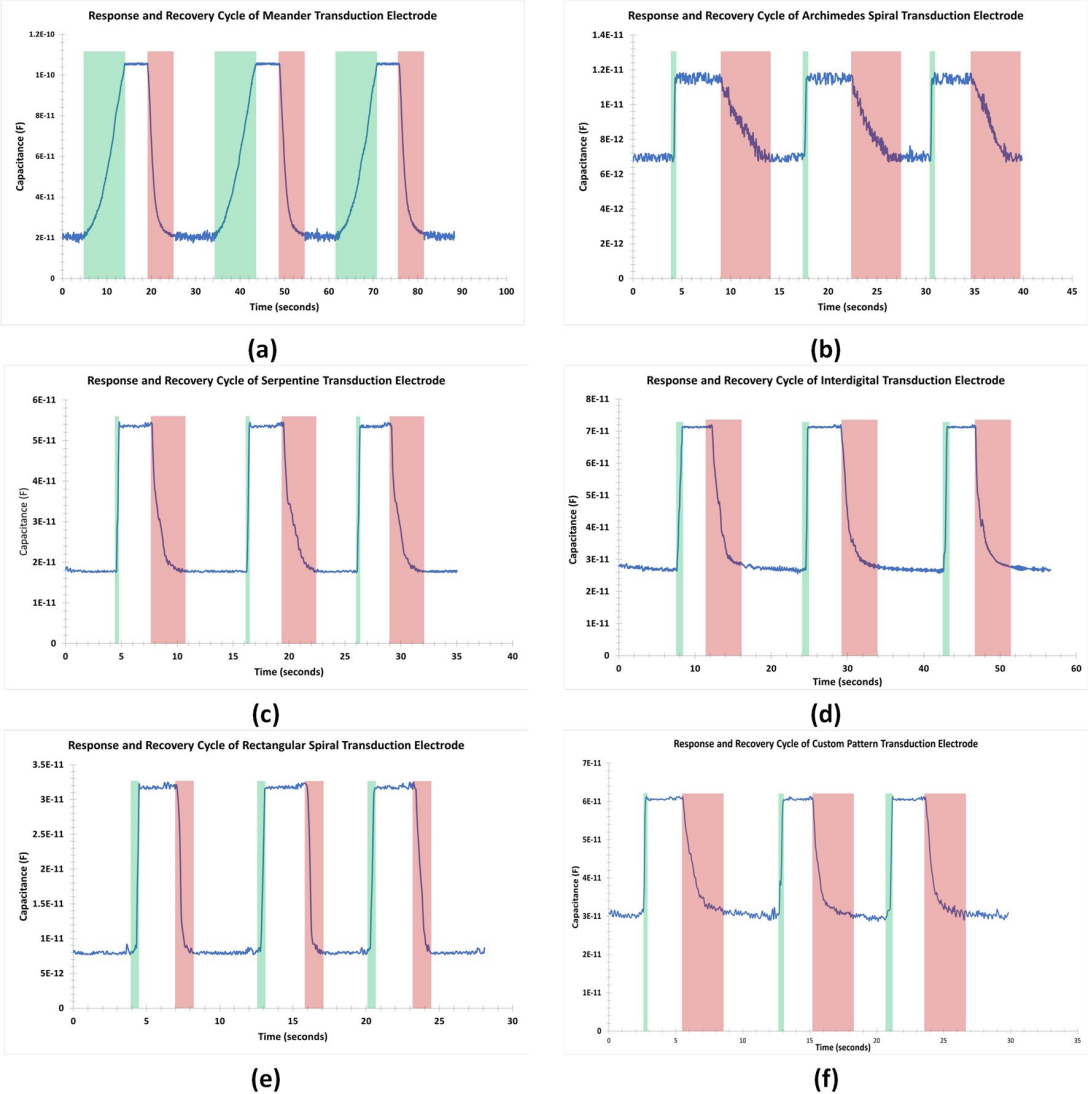
Response and Recovery cycle of patterned sensors for (**a**) Meander, (**b**) Archimedes spiral, (**c**) Serpentine, (**d**) Interdigital, (**e**) Rectangular spiral, and (**f**) Custom design.

Table 4 and Fig. 7 show the response and recovery cycles of all the patterned sensors. For each cycle of response time and recovery time of the sensor are calculated. The response time is highlighted in green and the recovery time is in red. Except the meander geometry all the other fabricated sensors have response time of less than 1 s, illustrating a quick humidity sensing application. However, the overall recovery times were below 6 seconds for all the geometries. A closer inspection of the bin sensitivities of all the transduction electrodes, as shown in Fig. 6, indicates that the meander transduction electrode geometry has exceptionally high sensitivity in the humidity bin of 90–100% when compared with the other transduction electrodes. Due to this effect the overall sensitivity of the meander geometry is higher as compared to the other geometries. Considering this factor and the gradual increase of transduction response of interdigital, serpentine, rectangular and custom geometries, it is eviden that they are well suited for humidity sensing in our case.

**Table 4:** Response and recovery of the transduction geometries.

Response and recovery of fabricated sensors		
Transduction geometry	Response (s)	Recovery (s)
Meander	9.38	5.90
Archimedes spiral	0.57	5.10
Serpentine	0.38	3.13
Interdigital	0.91	4.74
Rectangular spiral	0.57	1.28
Custom	0.54	3.11

**Figure 8:**
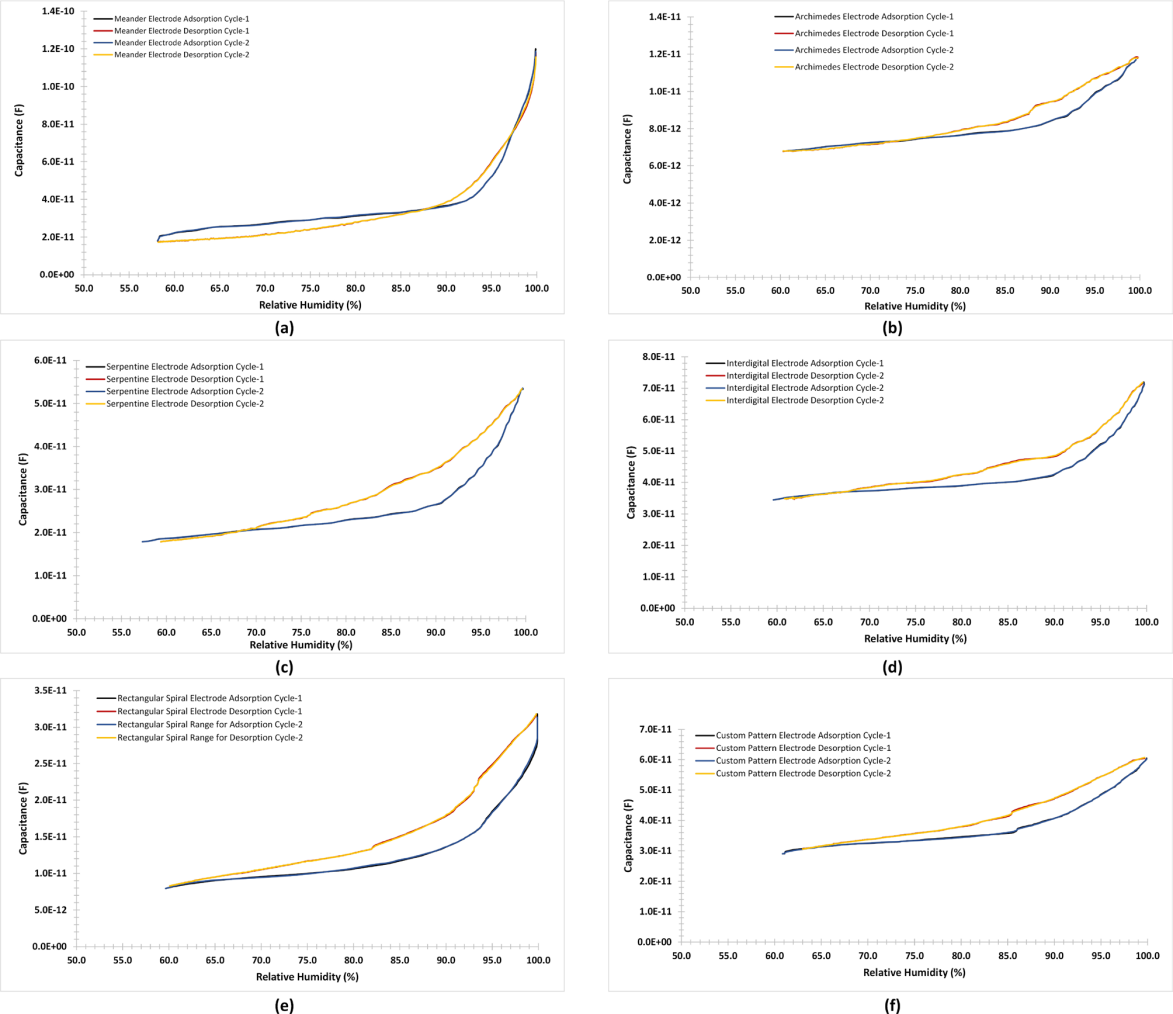
Adsorption and desorption cycle of patterned sensors for (**a**) Meander, (**b**) Archimedes spiral, (**c**) Serpentine, (**d**) Interdigital, (**e**) Rectangular spiral, and (**f**) Custom design.

Figure 8 provides the adsorption and desorption cycle of patterned sensors. We found that there exists a hysteresis between the adsorption and desorption cycle of the patterned electrodes in all the geometry. However, for certain geometry there the hysteresis is small compared to other geometries. The reason for the hysteresis is due to two factors. The first is due to the porous nature of cellulose layers on the sensing layer, which traps the water molecules during the desorption cycle. It is evident that the capacitive response for the desorption is more than the adsorption cycle of depicting the high chances of trapping water molecules.

The other reason is the gradual decrease of humidity level in the desorption cycle as compared to the adsorption cycle, which has a steep change in the humidity level. Since, the response of the DHT22 sensor has a higher rise due to the sudden increase of humidity in the chamber therefore, there is more hysteresis in the response stage of the sensor as compared to the recovery stage, where the hysteresis is low since the chamber humidity during this stage has a slower rate. It is anticipated that a precise measurement chamber can reduce the hysteresis between the response and recovery stage of the screen-printed sensors. It can be noted that in most of the cases the chamber starting humidity was a little higher at the end of the reading and the screen-printed response was also a little higher in the end depicting that there exists high correlation of humidity sensing of the sensors even with the slight deviation of humidity levels.

In the preceding discussion the results of the sensitivity, hysteresis, response and recovery of various transduction scheme was presented for humidity sensing. It is pertinent to note here that the sensitivity of the meander electrode was higher among all the fabricated sensors however, the response and recovery cycle was the lowest among all the geometries. Moreover, there was an appreciable increase in the capacitance value above 80% relative humidity, which indicates highly non-linear relationship between the humidity and respective capacitive response. In this regard, the most promising electrode configuration seems to be either serpentine and custom pattern as these were the only geometries, which provided a good sensitivities and less non-linearity among all the fabricated geometry.

## Methods

### Fabrication of transduction electrodes

**Figure 9:**
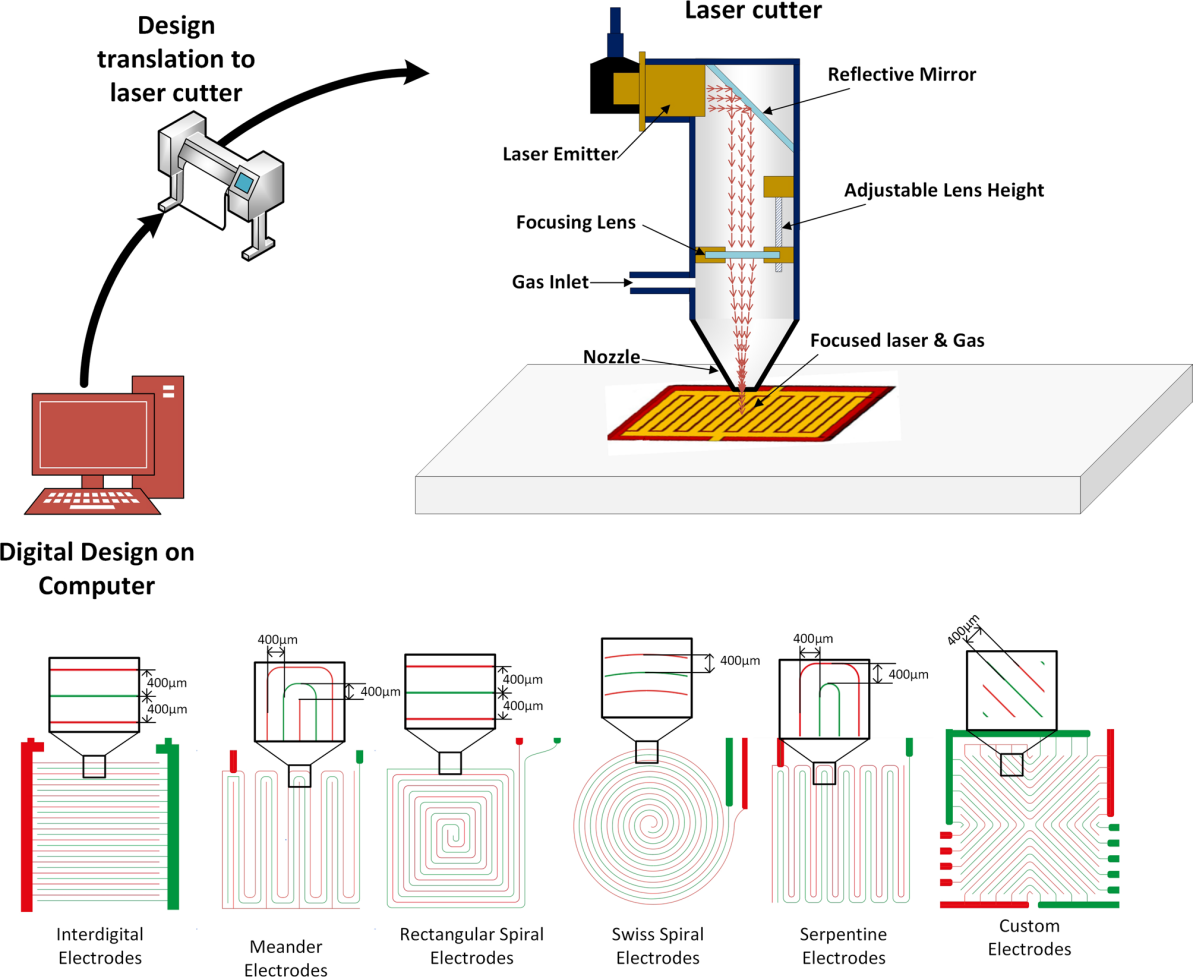
Transduction electrode fabrication process and transduction geometries.

Laser scribing is a method to induce high laser power to produce features or cuts on the surface of the substrate. In a mechanical workshop, the laser machine is used for cutting various materials of different thickness to perform 2D cutting and engraving. The main purpose is to transform the digital design to follow a laser path, which can be used for either cutting or engraving purposes. The depth of the cut depends upon the settings of power of the laser, speed of the laser and whether the spot size of the laser is focused on the substrate. On the other hand, the width of the cut depends upon the focusing lens, laser spot speed, power of the laser and distance of the laser with the object. If the laser is adjusted so as to focus properly on the substrate, then the quality of the laser cut is precise and is slightly above the focused spot size of the laser. The power of the laser is converted into heat energy when focused on the substrate and removes the material by locally ablate or burn the material to induce the digital imprints on the substrate. In the laser cutting process, the cut width is often termed as the kerf width of the laser cutting process. In order to reap the benefits of the above-mentioned process, we followed a facile process of engraving the designed pattern on the Poly (methyl methacrylate) (PMMA) sheets of thickness 3 mm. The printing process involves the computer-aided (CAD) designs of the electrode geometry and digitally transforming those through $\hbox {CO}_{\mathrm{2}}$ laser cutting beam on the PMMA sheets. Figure 9 highlights the overall process of fabrication.

The laser ablation was done by considering the glass transition temperature of the polymer substrate, laser speed, laser power and z-height of the laser beam without the optimization of the kerf width. This technique provides a rapid production of custom geometrical transduction scheme on the substrate. Due to the nature of the process, it requires no special micro fabrication process and can be done in a workshop environment to produce the transduction electrodes. Moreover, as the polymer becomes flexible close to its glass transition temperature, there is a possibility to adhere the substrate to curved surfaces. Moreover, the geometrical designs can be readily exported to the laser cutter, where the laser can be turned on and off instantly and the engraving/cutting features can be set for various layers. However, the limitation of the above technique is the resolution of the laser beam and the accuracy of stages of the $\hbox {CO}_{\mathrm{2}}$ beam laser cutter. In our experiments we used six different geometrical features all with line spacing of 400 $\upmu$m from the centre of the finger. Once the geometrical features were engraved on the PMMA sheets the edges of the sheets were cut out and a template for screen printing of conductive ink is ready for the next stage of conductive ink coating process. In our experiments we used the Novacentrix Metalon HPS-021LV (NOVACENTRIX, USA) screen printing ink. HPS-021LV is an electrically conductive silver flake ink designed to produce conductive traces on substrates such as paper, PET, glass, polyimide, and silicon. The main properties of HPS-021LV ink are listed in Table 5.

**Table 5:** Properties of HPS-021LV screen printing ink.

Measure	Value
Average particle size	2–4 $\upmu$m
Viscosity	26,000 cP at 0.1 s$^{-1}$
Specific gravity	3.1
Silver loading	75%
Solvent	Water

Once an ample amount of HPS-021LV was coated on the PMMA substrate the ink settles insides the engraved geometrical features. Later these geometrical features were subjected to heating in a convective oven to evaporate the solvent at 100 $^{\circ }$C, which is below the glass transition temperature of the PMMA sheet of 105 $^{\circ }$C. The thermal curing of the ink was done for 1 h each for all the geometrical features. After the curing process the sheet was cooled down to the ambient temperature and the excess ink was removed by uniformly scribing the surface of the PMMA sheets using a scribing knife. As the engraved features were below the level of the PMMA sheets, therefore, after the scribing process, only the ink necessary for forming the transduction electrode was left behind resulting in the functional sensing schemes. After the scribing process the conductivity of the tracks were checked through continuity measurement via a multimeter. Since, for each type of geometrical design the track lengths were different from the connection pad, the conductivity of the tracks varied for each geometrical feature.

### Sensing layer preparation

**Figure 10:**
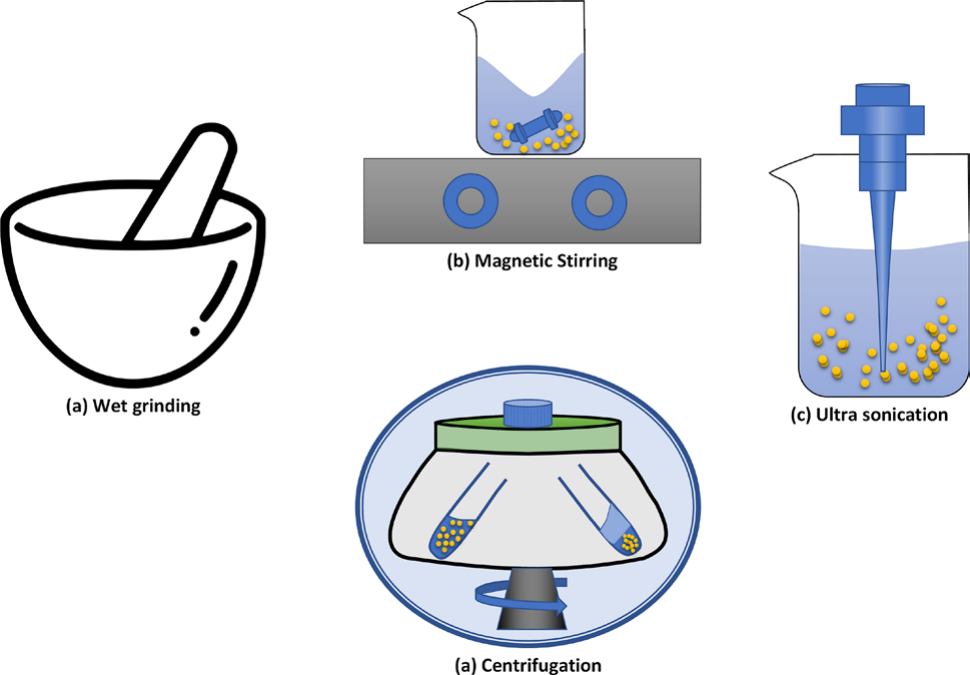
Process of ink preparation.

The process of ink preparation involves the synthesis of ultrafine particles from an amorphous precursor. For this purpose, a comprehensive methodology has been devised. The methodology involves the following steps as shown in Fig. 10.

The process starts with the wet grinding of 1 g of Sigmacell Cellulose (Product Code: S3504) of Type 20 with 20 $\upmu$m average diameter size with 5 ml of deionized water. The wet process improves the overall particle size by reducing the lumps and agglomerations occured during the storage of the cellulose. Shearing forces reduce the particles’ size, thus increasing the particles per unit weight. The reduction in particles increases the activation sites. During the 2 h grinding process the reduction in particle size in the mortar is felt with a decrease in friction of the grinding. Wet grinding was assisted by gradually adding water to maintain the solvent quantity during the process.

Later the mixture was transferred into the beaker and was weighed to record the concentration of solvent in the mixture. Once the weight measurement was taken, 50 ml of deionized water was added so facilitate the stirring process through a magnetic stirrer. Magnetic stirring of mixture provided a uniform homogenization of amorphous solid particles in the solvent. Thus, improving the uniformity of the suspended particles in the solution. After the stirring process the mixture was heated to 100 $^{\circ }$C to attain the solvent quantity of 5 ml after the evaporation process. A 1 ml of high conductivity poly(3,4-ethylenedioxythiophene)–poly(styrenesulfonate) (PEDOT:PSS) purchased from Sigma Aldrich (Product Code: 900181) having concentration of 0.5–1 wt% of PEDOT:PSS in water along with 0.1 ml of polyvinylpyrrolidone (PVP) coated silver nanoparticle of concentration 5 mg/ml in water (PVP-coated AgNP) purchased from NanoComposix was added to adjust the conductivity and to provide the steric stability of the mixture thus avoiding agglomeration of the suspended particles. The mixture was then probe sonicated twice for an interval of 5 min each to attain the homogenized mixture of cellulose decorated conductive polymer. After sonication the liquid was then subjected to centrifugation at 800 rpm for 30 min to remove the heavier particles from the mixture by removing the supernatant from the solution. The mentioned process provided a uniform concentration of cellulose particles when compared to the filtration process. In the filtration process only particles above a certain size are removed from the liquid. The shape of the particles remains the same, whereas the above-mentioned method shapes the particles into flakes or nanorods. This two-dimensional feature is more responsive when used for gas sensing applications^48^. Once the mixture was prepared the solution was poured on the transduction electrode and each transduction acrylic plate was then spin-coated at 1000 rpm for 120 s for each type of transduction geometry.

### Measurement setup

**Figure 11:**
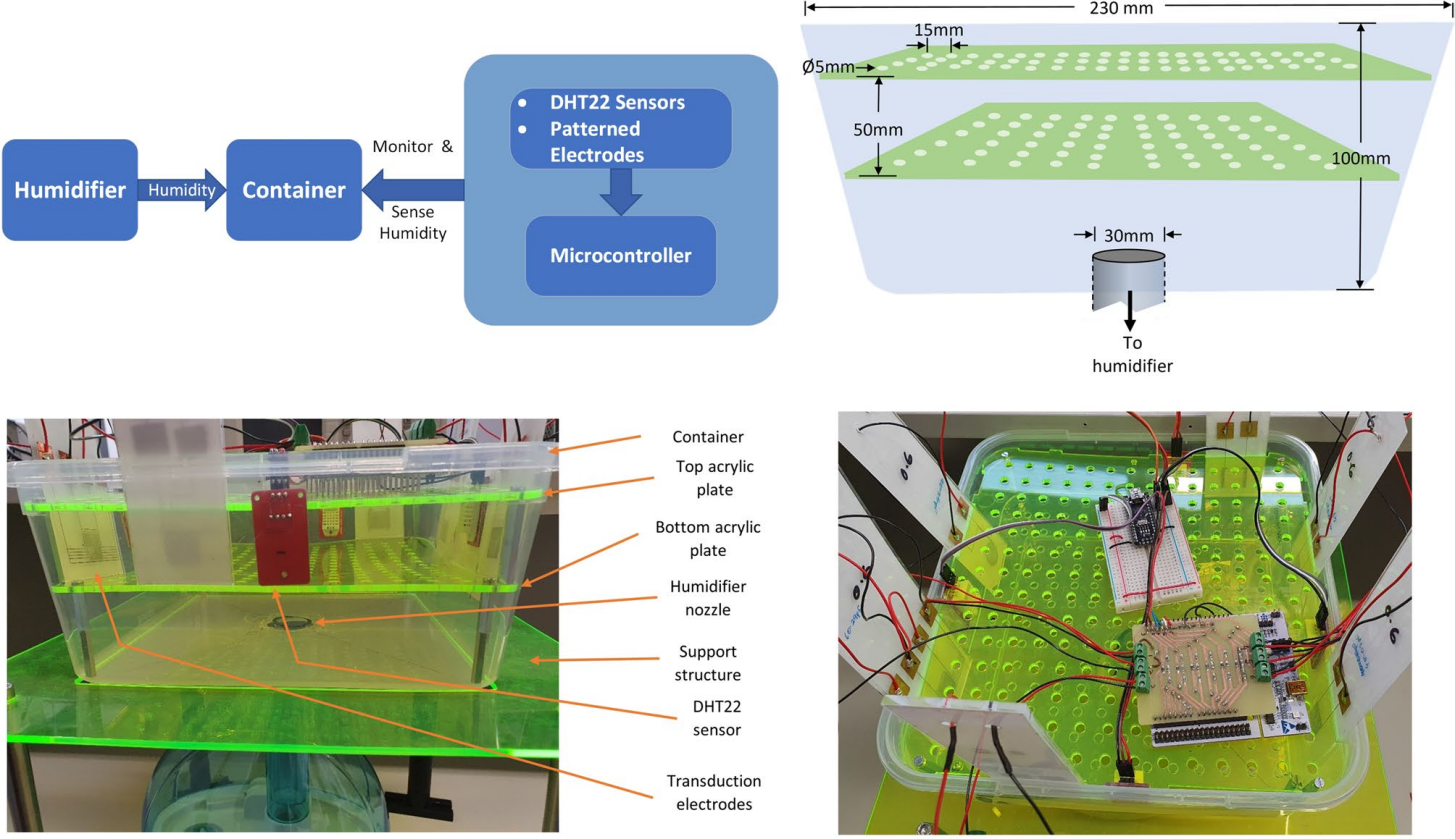
Layout of the experimental setup.

Transduction electrodes are commonly used in sensing applications. The electrodes provide the ability to measure different kinds of gases such as Nitrous oxide, gaseous Ammonia, humidity, and many more^49^. To test the performance of various patterns of electrodes, we chose to work with the most frequently measured physical quantity i.e. humidity. This provided us with a base for assessing performance parameters of various patterns of electrodes, built using in-house facilities. Since, the humidity level in an indoor setting is quite low and stable, we carried out the experiments in an environment where humidity could be controlled to monitor the behaviour of the electrodes. Therefore, an environment was built in a plastic container that was linked to an external humidifier where the level of humidity was varied and continuously monitored. The basic layout of the experimental setup is depicted in Fig. 11.

The container was built such that the DHT22 sensors and different patterns of electrodes could be placed inside it. To achieve homogeneity of humidity inside the container, two acrylic plates were placed horizontally inside the container with holes made using a laser cutter. The holes of 5 mm in diameter were spread evenly across both plates with 15 mm spacing between them, and a 7 mm offset in holes between the top and bottom plate. The bottom plate assisted in dispersing humidity evenly while the top plate assisted in releasing excess humidity out of the chamber. Four DHT22 humidity sensors were placed on all four sides of the container such that the sensors and the electrode sensors were placed vertically around the inner walls of the container located between the top and the bottom plates. This enabled us to simultaneously test the performance of all the different patterns of transduction electrode sensors with varying levels of humidity. The two plates with holes fit inside the container with a much larger hole cut out at the centre of the container for a plastic pipe that drops down to the humidifier.

Humidity can easily be generated using an appliance called the humidifier, which is inexpensive and provides the user with the ability to control the humidity. The humidifier used in the experiment is a droplet free ultrasonic cool mist generator  and has a 1.5 l water tank (Kogan Mini 1.5L Humidifier). The humidifier was placed at the bottom of the container with a circular hole cut out at the bottom for a tight fit of a 30 mm wide pipe connecting to the outlet of the humidifier. A support structure was built to place the container on top of the humidifier. The humidifier exudes mist from the top into the bottom of the container through a pipe. Each transduction pattern has two square pads filled with silver ink that extends to the sensing electrodes themselves. With the help of adhesive copper tape, small pieces were fixed to the two pads, so that the thin multistrand wires could be soldered on to the tape for connectivity. As soon as the humidifier starts introducing humidity into the container, DHT22 sensors begin reading humidity levels and simultaneously the transduction electrodes start sensing the humidity levels. Humidity levels from DHT22 sensors, were read using Arduino Nano that was kept separate from the capacitive readings taken with Nucleo-F446RE board. Components of the experimental setup are labelled in Fig. 11.

The top view of the experimental setup indicates the position of the transduction electrodes of six different patterns (meander, interdigital, serpentine, circular spiral, rectangular spiral and a custom design) and four DHT22 humidity sensors, all mounted on inner walls of the container.

## Conclusion

In this work, we presented a comparison among six different electrode layouts fabricated by using a laser ablation process. It has been observed that depending on the specific application and its requirements, an appropriate transduction scheme for environmental humidity sensors can be ascertained. For a large area sensing applications, the presented designs are scalable and suitable for sensing applications. The custom triangular pattern presented in this work can be a promising scheme when scalability for large area is not an issue. The fabricated sensors were tested at various relative humidity levels that achieved a good sensing response with sensitivities ranging from 0.13 to 2.37 pF/%RH in general for various transduction schemes. The meander geometric transduction scheme reported the highest sensitivity among the fabricated sensors however, there were some demerits for this geometry, such as lower response and recovery time along with associated non-linearity of capacitive response with respect to humidity. The work presented here provides a facile approach, biocompatible sensing layer and compendium of processes for fabricating sensors in a small low-cost laboratory, which can be of great advantage during the prevailing COVID-19 pandemic. Furthermore, the results obtained from the presented fabrication scheme can be extended for a high-resolution patterning electrode geometry with a suitable sensing layer.
